# Not All Factors Contribute Equally to European-American and Hispanic Students’ SAT Scores

**DOI:** 10.3390/jintelligence7030018

**Published:** 2019-08-01

**Authors:** Brenda Hannon

**Affiliations:** Department of Psychology & Sociology, Texas A&M University-Kingsville, Kingsville, TX 78363-8202, USA; brenda.hannon@tamuk.edu; Tel.: +361-593-2698

**Keywords:** cognitive factors, SAT, SAT-V, SAT-M, test anxiety, performance avoidance, SES, ethnicity, Hispanics, students

## Abstract

This exploratory study shows that the contributions of cognitive, metacognitive awareness, performance avoidance, test anxiety, and socioeconomic family background factors to SAT scores (i.e., overall SAT, SAT-V, SAT-M) may vary as a function of ethnicity (i.e., European-American, Hispanic). Four hundred and fifty-seven students, 282 European-American and 175 Hispanic, completed multiple measures of cognitive, metacognitive awareness, social/personality (i.e., test anxiety, performance avoidance, academic self-efficacy), and socioeconomic family background factors, which were used in regression analyses predicting overall SAT, SAT-V, and SAT-M scores. The results show that most factors contributed significantly to overall SAT, SAT-M, and SAT-V scores. In addition, the ethnicity X test anxiety interaction was significant for all three SAT measures, a finding that suggests ethnic differences in the contributions of test anxiety to overall SAT, SAT-M, and SAT-V scores. For European-American students, test anxiety had no influence on overall SAT and SAT-M scores, whereas for Hispanic students test anxiety had a negative influence on overall SAT and SAT-M scores. For SAT-V scores, interpreting the ethnicity X test anxiety interaction was more complicated because both the significant main effect of test anxiety and the ethnicity X test anxiety interaction must be interpreted together. Whereas test anxiety negatively influenced European-Americans’ SAT-V scores, this negative influence was less than the influence it had on Hispanic students’ SAT-V scores. Indeed, for Hispanic students with high test anxiety, this negative influence was profound. Taken as a whole, these results suggest that any theory explaining the SAT may need to take into account multiple predictors as well as the possibility that the contributions of these predictors may vary as a function of ethnicity.

## 1. Introduction

According to a recent survey, nearly all institutions of higher education in the United States consider scores on the SAT (formerly called the Scholastic Assessment Test and The Scholastic Aptitude Test but is now just called the SAT) to be of “considerable” or “moderate” importance in college/university admissions [1,2]. So it is not surprising that more than 1.5 million college bound students complete the SAT each year [3]. Yet, as important as the SAT is, uncertainty about its construct validity and large gender and ethnic differences still persist. It is this latter concern—ethnic differences in SAT scores—that is of primary interest to the present paper. More specifically, the present study explored the contributions of non-cognitive factors, to European-American and Hispanic students’ overall SAT, SAT-V (i.e., verbal section of SAT), and SAT-M scores (i.e., math section of SAT) after the contributions of cognitive factors (e.g., knowledge integration, working memory), were removed. The non-cognitive factors examined here were: (i) metacognitive awareness, which pertains to beliefs about knowledge and learning, (ii) social/personality factors, which are factors that affect personality, attitudes, and/or lifestyle (e.g., test anxiety, performance avoidance, academic self-efficacy), and (iii) socioeconomic family background factors. See Crede’ and Kuncel who separate cognitive, metacognitive, social/personality, and socioeconomic family background into cognitive and non-cognitive (i.e., social, personality, socioeconomic family background) research areas [4]. The present study adopts Crede’ and Kuncel’s terminology of cognitive versus non-cognitive (i.e., factors that do not assess cognitive abilities/processes) [4]. The present study also includes metacognitive awareness as non-cognitive^1^. Measures of overall SAT, SAT-V, and SAT-M were selected because US universities use one, two, or all three of these measures during admissions. Below is a brief review of the literature regarding ethnic differences and the overall predictors of overall SAT, SAT-V, and SAT-M scores. In the final section is a description of the present exploratory study that examines the contributions of metacognitive awareness, test anxiety, performance avoidance, academic self-efficacy, and socioeconomic family background factors to European-American and Hispanic students’ overall SAT, SAT-V, and SAT-M scores after the contributions of cognitive factors have been removed. That is, do metacognitive awareness, test anxiety, performance avoidance, academic self-efficacy, and socioeconomic family background factors contribute to European-American and Hispanic students’ SAT scores to the same extent?

### 1.1. Ethnic Differences in Overall SAT, SAT-V, and SAT-M Scores

Centermost to the ethnic disparity debate is that “racial/ethnic minority students (i.e., Hispanic and African-American) continually score lower on SAT measures than do non-minority students (i.e., European-American) [2,5] and these racial/ethnic differences have persisted for nearly 50 years [6]^2^. Indeed, 2015 data, which include all senior, college-bound SAT test takers, indicate that Hispanic students’ mean SAT-V and SAT-M scores (448 and 457 respectively) were consistently lower than European-American students’ mean SAT-V and SAT-M scores (529 and 534 respectively) [7]. Furthermore, data, which include all senior college-bound Hispanic and European-American SAT test takers, for the 20-year period from 1987 to 2006, suggest a similar pattern of results, with mean SAT-V and SAT-M scores for Hispanic students (456 and 460 respectively) consistently lower than mean SAT-V and SAT scores for European-American students (526 and 523 respectively) [6]^3^.

Yet, despite both the magnitudes of these differences and their implications, few studies have attempted to identify the sources of ethnic differences in SAT scores [2,8,9]. Moreover, the bulk of the studies that have attempted have had limited success. For instance, although studies have suggested stereotype threat [10] and variability in educational opportunities as sources of ethnic differences in SAT scores, stereotype threat and variability in educational opportunities contribute only in small ways to ethnic differences in SAT, SAT-V, or SAT-M scores [2]. Furthermore, some researchers consider stereotype threat to be an unreliable source of variance [11].

A recent study by Hannon [2], however, has provided some valuable insights into some of the sources of ethnic differences in SAT, SAT-V, and SAT-M scores. In Hannon’s [2] study, European-American and Hispanic students completed two measures of socioeconomic family background factors (i.e., parental education and family income), test anxiety, performance avoidance goals, and a measure of metacognitive awareness. The contributions of these measures were then systematically controlled in a series of analysis of covariance tests that assessed ethnic differences in SAT, SAT-V, and SAT-M scores. The results revealed that parental education, performance-avoidance goals, and metacognitive awareness significantly contributed to the ethnic disparity in SAT, SAT-V, and SAT-M scores. More specifically, Hannon [2] observed that when parental education, performance-avoidance goals, and metacognitive awareness were controlled, the effect sizes for ethnicity (i.e., ethnic differences) on SAT, SAT-V, and SAT-M scores decreased by 55 to 75%.

As informative as Hannon’s [2] study is, it does, however, have some limitations. For instance, Hannon’s [2] study did not examine the contributions of the measures of performance avoidance, test anxiety, socioeconomic family background (i.e., parental education, family income), and metacognitive awareness to overall SAT, SAT-V, and SAT-M scores of European-American versus Hispanic students. That is, Hannon did not determine whether these measures contributed to European-American and Hispanic students’ SAT scores to the same extent. However, even more importantly, Hannon’s study did not include measures of cognitive abilities, which routinely account for a considerable amount of variance in SAT, SAT-V, and SAT-M scores [12,13,14,15]. Thus, it is unclear what contributions cognitive factors versus metacognitive awareness, test anxiety, performance avoidance factors, and socioeconomic family background factors make to overall SAT, SAT-V, and SAT-M scores for European-American versus Hispanic students.

### 1.2. Predictors of the SAT, SAT-V, and SAT-M

Over the years, a number of studies have identified factors that influence or explain individual differences in SAT scores; however, the factor(s) under investigation frequently varies from study to study [15]. In Frey and Detterman’s [12] seminal work, which included more than 11,500 students, multiple intelligence measures (e.g., the Coop School and College Ability Test, the California Test of Mental Maturity, the Armed Services Vocational Aptitude Battery, Ravens Advanced Progressive Matrices, and the Otis-Lennon Mental Ability Test) were correlated separately with overall SAT scores. Frey and Detterman [12] observed strong, across-the-board correlations between all the measures of intelligence and SAT scores, *range of r* = 0.53 to 0.82—a finding that led them to suggest that the SAT is primarily a measure of general intelligence or “g” [14]. From a theoretical perspective, presumably cognitive measures, such as general intelligence, are excellent predictors of SAT scores because both intelligence measures and SAT scores involve continuous problem solving [14].

Other cognitive factors also predict the SAT. For example, working memory, which is an individual’s capacity to simultaneously process and store information, is a strong predictor of SAT, SAT-V, and SAT-M scores (e.g., [16,17,18]). Indeed, research suggests that complex measures of processing + storage, like working memory measures, are much stronger predictors of SAT scores than are the traditional short-term memory storage-only measures [18]. Additionally, knowledge integration, which is an individual’s ability to integrate prior knowledge with new text-based information, is also a strong predictor of SAT, SAT-V, and SAT-M scores [14,15].

Research also suggests that a number of social/personality factors predict SAT scores. For instance, test anxiety, which is typically characterized as a fear of failing that precedes an upcoming examination, routinely accounts for 10 to 17% of the variance in SAT scores [15,19,20,21]. Performance-avoidance, which is characterized as avoiding undesired outcomes on examinations, is inversely related to performances on exams [22] and typically accounts for 9 to 15% of the variance in SAT scores [13,15]. Academic self-efficacy, which is characterized as one’s belief in one’s ability to succeed, is also related to ACT/SAT scores [15,23,24]. Furthermore, socioeconomic family background factors (i.e., SES), such as the level of parental education and/or household income, are related to students’ SAT scores inasmuch as students who come from homes with higher levels of parental education and income tend to perform better on the SAT than their less fortunate counterparts [2,25]. Finally, metacognitive awareness, which is an individual’s knowledge/beliefs about the complexity of knowledge and value of knowledge integration, is also predictive of SAT, SAT-V, and SAT-M scores.

More recently, Hannon and McNaughton-Cassill [15] examined the simultaneous influences of cognitive, metacognitive awareness, test anxiety, and performance avoidance factors on SAT, SAT-V, and SAT-M scores. In their study, cognitive (i.e., working memory, knowledge integration), and metacognitive awareness measures, accounted for 37.8% of the variance in SAT scores, 41.4% of the variance in SAT-V scores, and 21.9% of the variance in SAT-M scores, while their social/personality measures of test anxiety and performance avoidance accounted for 21.4% of the variance in SAT scores, 18.2% of the variance in SAT-V scores, and 17.3% of the variance in SAT-M scores [15]. Moreover, when cognitive and social/personality measures were both included in the same regression models, they accounted for 43.4% of the variance in SAT scores, 44.6% of the variance in SAT-V scores, and 28% of the variance SAT-M scores.

As informative as Hannon and McNaughton-Cassill’s [15] study is, it is, nevertheless, not without its limitations. One notable limitation is the absence of SES, which is related to students’ SAT scores [2,25]. Another limitation is that Hannon and McNaughton-Cassill [15] did not identify European-American and Hispanic students as separate ethnic groups. Consequently, it is unclear whether cognitive, metacognitive awareness, test anxiety, performance avoidance goals, and socioeconomic family background factors contribute to SAT scores to the same extent for these two separate groups of students.

### 1.3. Current Study

In summary, previous research suggests that when parental education, performance avoidance, and metacognitive awareness are controlled, the effect size attributed to ethnic differences in SAT, SAT-V, and SAT-M scores decreases by 55 to 75% [2]. Previous research also suggests that cognitive, metacognitive awareness, and/or test anxiety and performance avoidance factors predict SAT, SAT-V, and SAT-M scores [11,13]. However, because the former study did not control for cognitive factors, it is unclear whether cognitive factors contribute to ethnic differences in SAT, SAT-V, and SAT-M scores. Moreover, because the latter study, which did include cognitive factors, failed to determine the relative contributions to SAT scores for European-American versus Hispanic students, it is unclear what are the contributions of cognitive, metacognitive awareness, test anxiety, performance avoidance goals and, socioeconomic family background factors to European-American versus Hispanic students’ SAT, SAT-V, and SAT scores. The present exploratory study combines the goals of this previous research by examining the contributions of cognitive, metacognitive awareness, test anxiety, performance avoidance, academic self-efficacy, and socioeconomic family background factors to overall SAT, SAT-V, and SAT-M scores for European-American versus Hispanic students. That is, are the contributions of these factors to overall SAT, SAT-V, and SAT-M scores the same or different for these two ethnic groups? And are the contributions of a cognitive factor greater than/less than or equivalent to factors that are not cognitive?

In order to answer these questions, the present study completed regression analyses that predicted overall SAT, SAT-V, and SAT-M scores. Each regression not only included measures of: (i) cognitive abilities (i.e., working memory, knowledge integration), (ii) test anxiety, (iii) performance avoidance, (iv) academic self-efficacy, (v) metacognitive awareness, (vi) socioeconomic status, and (vii) ethnicity but also included a number of interaction terms: (viii) ethnicity X cognitive abilities, (ix) ethnicity X test anxiety, (x) ethnicity X performance avoidance, (xi) ethnicity X academic self-efficacy, (xii) ethnicity X metacognitive awareness, and (xiii) ethnicity X socioeconomic status. These ethnicity X predictor interactions are particularly important to the present study because if any of these interactions are significant, it would mean that the contributions that cognitive, metacognitive awareness, test anxiety, performance avoidance, academic self-efficacy, and/or socioeconomic family background factors make to overall SAT, SAT-V, and/or SAT-M scores vary as a function of the ethnicity of a student. This analysis was completed by using two previously-created datasets. Subsets of data from dataset one were used in four other published studies [2,13,15,26], and a subset of data from dataset two generated one other published paper [14].

All of the measures that are used in the present study are measures that are both frequently-used and reliable. For the composite measure of cognitive abilities, Hannon and Daneman’s [27] knowledge integration measure, which assesses a reader’s ability to integrate prior knowledge with text-based information [13,27,28,29,30,31,32,33,34] and Turner and Engle’s [35] operation span, a combined processing+storage measure of working memory were used because previous research has observed high correlations between these measures [18,34]. Indeed, because of the routine high correlations between measures of working memory and general intelligence, some researchers [36,37,38] have asserted that working memory and general intelligence may be essentially the same construct [34], (pp. 176–177); although other researchers argue that it may be relational integration, an underlying component of working memory, that is the source of these high working memory-general intelligence correlations [34,39]. The measure of metacognitive awareness was Rukivina and Daneman’s [40] variant of Schommer’s [41] measure of epistemic belief of learning. The measures of socioeconomic status were highest parent education and family income. Finally, the measures of social/personality factors were Sarason’s [42] measure of test anxiety, Elliot and Church’s [22] measure of performance avoidance, which was a subtest of their achievement motivation measure, and McIlroy, Bunting, and Adamson’s [43] measure of academic self-efficacy. Although Hannon [2,14] did not include academic self-efficacy, academic self-efficacy is included here because other studies have shown that academic self-efficacy is a significant predictor of SAT, SAT-V, and SAT-M scores [23,24].

Based on the findings of previous research [2,13,14,18,19,20,21,22,24,35,36], it is expected that cognitive, metacognitive awareness, test anxiety, performance avoidance, academic self-efficacy, and socioeconomic family background factors will significantly contribute to students’ overall SAT, SAT-M, and SAT-V scores. What is uncertain is the extent to which these factors contribute to the SAT measures of European-American versus Hispanic students. That is, are the contributions of these factors the same or different for European-American versus Hispanic students’ overall SAT, SAT-M, and SAT-V scores? For example, perhaps test anxiety impacts Hispanic students’ SAT, SAT-M, and SAT-V scores much more than it does European-American students’ SAT, SAT-M, and SAT-V scores.

## 2. Materials and Methods

### 2.1. Participants

The 457 university students were a combination of students from two datasets. Three hundred and twenty-six students were from dataset one, which was a project funded by NIH (grant # 1 R24 MH070636—01A1) and paid students $40.00 for completing two sessions. To date, subsets of data from dataset one, which includes a total of 428 students, have generated four published papers [2,13,15,26]. The 326 students that were included in this study were all of the students in dataset one who had both completed the SAT and were of European-American or Hispanic descent (i.e., there were other students in dataset one but they had completed just the ACT, did not complete either the SAT or ACT, had not completed the full study, or spoke too much of another language other than English). The 131 students from dataset two, which included a total of 237 students, received $70.00 for participating in a study that examined the neural and cognitive components of reading. To date, dataset two has generated one published paper [13] that included 171 students. However, because 40 of these 171 students could not be classified as either European-American or Hispanic, they were excluded from the present study. The remaining 66 students in dataset two were also excluded from the present study because they had not completed the SAT or had not completed all the measures used in the present study.

All students gave their informed consent for inclusion before they participated in these studies. These studies were conducted in accordance with the Declaration of Helsinki, and the protocols were approved by the Ethics Committee of UTSA (05-398; 09-164).

Students were 18 to 27 years old, *M* = 19.32 (*std* = 1.53), and were pre-screened up to two weeks prior to participating in either study described above. The pre-screening was completed in order to ensure that: (i) he/she did not suffer from a learning disability, (ii) he/she was a dominant English speaker, and (iii) to learn about his/her availability for scheduling purposes. If a student suffered from a learning disability and was not a dominant English speaker, he/she was not invited to participate in either of the two studies described above. Two hundred and fourteen of the students were male and 243 of the students were female.

Two hundred and eighty-two of the participants were of European-American descent and 175 of the participants were of Hispanic descent. Ethnicity was determined by asking the following questions: (i) Which ethnic group do you identify with? _________, (ii) What is the ethnic background of your mother? ________, (iii) What is the ethnic background of your father? __________. Consistency among the answers was necessary in order to be included in the present study. In instances of ambiguity (e.g., student says ancestors are from Florida), additional questions were asked until the ethnicity could be determined. If a student’s ethnicity was still undetermined, he/she was classified as either: (i) mixed if he/she had more than one ethnic background, or (ii) unknown if both parents’ ethnicities could not be identified. Although students of mixed or unknown ethnicities were invited to participant in the study for dataset two (but were excluded in the present study), students of mixed or unknown ethnicities were not invited to participate in the study for dataset one because this study required clear ethnic classifications. Finally, although 457 students were originally included in this study, the data for two students were removed as a consequence of the data screening process detailed in the results section.

### 2.2. Measures

Because all of the measures used in the present study have been used in many other published studies, the present study provides only brief descriptions.

#### 2.2.1. SAT Scores

All students consented to the release of their SAT scores from university records. Overall SAT scores were calculated by summing SAT-V and SAT-M scores. All SATs were completed after 2008 and before 2011. According to the College Board [44,45], the SAT, SAT-V, and SAT-M each have a 0.91 reliability (at minimum).

#### 2.2.2. Measures of Social/Personality Factors

Three social/personality factors were assessed: performance avoidance, test anxiety, and academic self-efficacy. See Elliot and Church [22], Sarason [42], and McIlroy et al. [43] for details about these measures. All three of these measures were presented on a computer. For each measure, a single item appeared on the computer screen until the student responded. At this point, the item disappeared and the next item appeared. The following brief descriptions of these measures are paraphrases from Hannon [2] and Hannon and McNaughton-Cassill [15].

The performance avoidance measure was a subscale of Elliot and Church’s [22] achievement motivation measure. There were six items and each student selected his/her answer using a 7-point Likert scale. A sample item is: *I worry about the possibility of getting a bad grade in my classes.* A high score indicates that a student has a high propensity towards avoidance during/before upcoming periods of assessment. The Cronbach alpha for this measure in this study was 0.71.

The test anxiety measure was developed by Sarason [42]. This scale includes 37 true–false statements with high scores indicating greater test anxiety. However, because of a computer malfunction the measure was reduced to 36 items for each participant. A sample item is *I have an uneasy, upset feeling before taking a final exam.* The Cronbach alpha for this measure in this study was 0.90.

The measure of self-efficacy was McIlroy et al.’s [43] academic self-efficacy scale. McIlroy et al.’s [43] self-efficacy scale includes 10 items and each item includes a 7-point Likert scale. A sample item is: *All and all, I feel that I am largely in control of my exam outcomes.* High scores on this measure indicate high self-efficacy. The Cronbach alpha for this measure in this study was 0.81.

#### 2.2.3. Measures of Cognitive and Metacognitive Awareness Factors

Students completed two measures of cognitive abilities: the operation span and knowledge integration. See Daneman and Hannon [46,47] and Turner and Engle [35] for details about the measure of working memory and Hannon and Daneman [27] and Hannon [13,15] for details about the measure of knowledge integration. A composite z-score of cognitive abilities was calculated by summing together the z-scores for the operation span and the knowledge integration measure. Students also completed one measure of metacognitive awareness, namely Schommer’s [41] epistemic belief of learning.

The operation span consists of increasingly longer sets of equations and words that are presented on a computer. Students read aloud the math equation and word, decide whether the math equation is correct/incorrect, and then at the end of the set, which is signaled by a blank blue screen, recall the words. For example, for the two-item set *6 × 3 = 7 table; 2 + 1 = 3 radio*, a student will read aloud the math equation and word: *6 × 3 = 7 table,* decide whether the math equation is correct/incorrect, press the spacebar for the next equation: *2 + 1 = 3 radio,* read this equation aloud, decide whether the equation is correct/incorrect, press the spacebar, and then at the blank blue screen recall the words: *table, radio*. The total number of words recalled correctly is the dependent measure. Higher scores indicate a greater working memory capacity. The maximum score is 100. The Cronbach’s alpha for this measure in this study was 86.

The knowledge integration measure was a subtest of a multi-component task called the component processes task (i.e., CPT) [27,31,32,33,34]. The brief description below is a paraphrase of a number of papers [2,14,30,31].

In the CPT, students learn passages that consist of three sentences, which are presented individually in the middle of a computer screen. For example: *A MIRT resembles an OSTRICH but is larger and has a longer neck.; A COFT resembles a ROBIN but is smaller and has a longer neck.; A FILP resembles a COFT but is smaller, has a longer neck, and nests on land.* After reading the passage, true–false statements that assess four component processes, namely text memory, text inferencing, knowledge access, and knowledge integration, appear individually in the middle of a computer screen. Of primary interest to the present study are the knowledge integration statements, which assess a person’s ability to integrate text-based information with information from long-term memory. For example, for the above bird paragraph, the knowledge integration statement “*Like PENGUINS, MIRTS don’t fly*” requires a student to access knowledge about birds from his/her long-term memory (e.g., *that some birds, like penguins don’t fly*) and integrate this knowledge with new the text-based information (e.g., *that MIRTS resemble OSTRICHES and consequently do not fly*). As this example shows, knowledge integration statements include a nonsense term that appeared in the paragraph (e.g., *MIRTS*) and a real term (e.g., *PENGUINS*) and a semantic feature (e.g., *can’t fly*) that do not appear in the paragraph. In total, there were 12 knowledge integration statements and higher scores indicate better knowledge integration ability. The Cronbach alpha for this measure in this study was 0.91.

Finally, the pencil and paper measure of metacognitive awareness was 12 items selected from two subtests of Schommer’s [41] epistemology questionnaire; see [15] for an identical questionnaire and administration. For each of the 12 items, students select their level of agreement using a 5-point Likert scale [14]. A sample item is: *Being a good student generally involves memorizing facts*. High scores on this measure are indicative of mature beliefs about learning and low scores are indicative of naïve beliefs about learning. The Cronbach alpha for this measure in this study was 0.64.

#### 2.2.4. Measures of Socioeconomic Status

Two measures of socioeconomic status were assessed: (i) the education level of the parent who had attained the highest education level and (ii) family income [48]. Although the education levels of both parents were assessed using a scale from 1 to 9, where 1 represented less than high school and 9 represented post-doctoral education, only the highest parent education was used; see [49,50] for discussion of this point. For family income, the measure ranged from 0 to 7, where 0 was <$12,000/year and 7 was $100,000+/year [2]. A composite z-score of socioeconomic status (i.e., SES) was calculated by calculating z-scores for highest parent education and family income and adding these two z-scores together.

## 3. Results

The results are reported in four sections: (i) data screening, (ii) the descriptive statistics and correlational analysis for the group data, (iii) the descriptive statistics and correlational analysis for each ethnic group (i.e., European-Americans, Hispanics), and (iv) the regression analyses that determined the contributions of cognitive, metacognitive awareness, test anxiety, performance avoidance, academic self-efficacy, and socioeconomic predictors to overall SAT, SAT-M, and SAT-V scores as a function of ethnicity. Where necessary, two-tailed t- and z-tests were computed. Significance levels were set to 0.05 unless otherwise specified. SAS 9.4 was used to complete data screening, correlational analysis, and regression analysis. Microsoft Excel was used to calculate t-tests and z-tests.

### 3.1. Data Screening

Three regression analyses—one for each SAT measure—were used to generate the following screening statistics: (i) outliers (i.e., studentized residuals, DFITTs, Cook’s Ds; DFBETAs), (ii) data points that exerted excessive leverage (i.e., *h*, also known as the hat value), (iii) linearity (bivariate scatterplots), (iv) normality (i.e., normality probability plots), (v) heteroscedasity (i.e., White’s test), and (vi) multicollinearity of predictors (tolerance test). Each regression analysis included the predictors identified in Table 1, Table 2 and Table 3 as well as all other non-significant predictors^4^.

**Figure 1:**
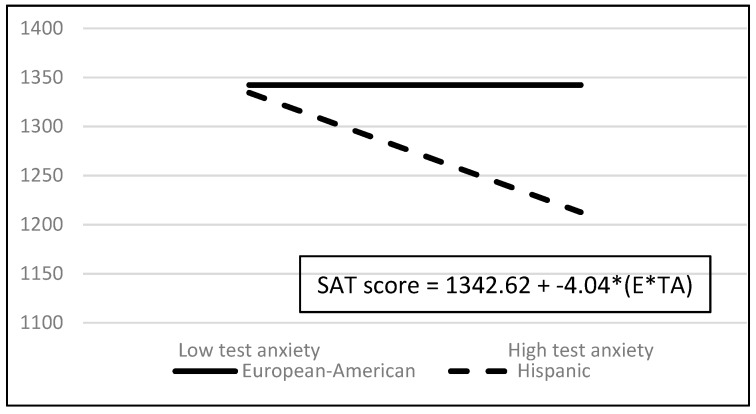
Overall SAT scores as a Function of the Ethnicity X Test Anxiety Interaction. E * TA = ethnicity X test anxiety interaction.

**Figure 2:**
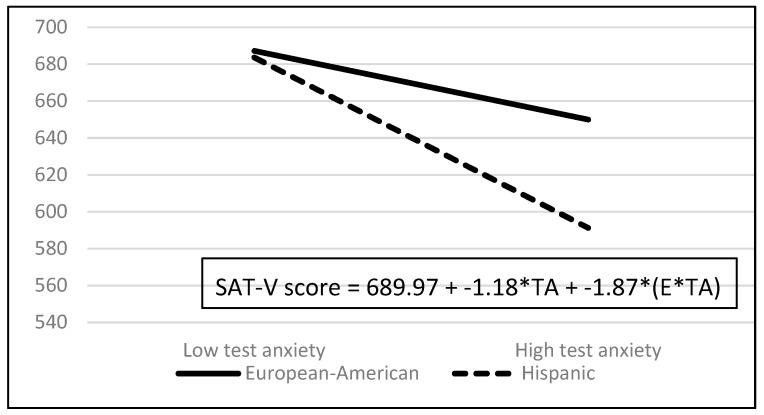
SAT-V scores as Function of the Test Anxiety Main Effect and the Ethnicity X Test Anxiety Interaction. TA = main effect for test anxiety and E * TA = ethnicity X test anxiety interaction.

Examination of the screening statistics revealed two outliers (i.e., studentized residuals, DFITTs, Cook’s Ds, and DFBETAs were beyond acceptable limits). Data for these two outliers were removed and the three regressions were repeated using the data for the remaining 455 students. The results of the second set of screening statistics revealed no outliers (i.e., studentized residuals, DFITTs, Cook’s Ds, and DFBETAs were all within acceptable limits), no points with excessive leverage, linearity, normality, no hetroscedasity (i.e., all White’s tests had *p*’s > 0.10), and no multicollinearity (i.e., tolerance values all well above the 0.20 threshold). The data for the two outlying students were permanently removed and consequently all analyses reported below and numbers reported in Table 1, Table 2 and Table 3 and Figure 1 and Figure 2 exclude the data from these two outlying students.

### 3.2. Descriptive Statistics and Correlations for Group Data

Table 1 reports the descriptive statistics (i.e., means, standard deviations, skewness, kurtosis, ranges, and maximum scores) and correlations among the measures. As Table 1 shows, all measures had large ranges—a finding that suggests the absence of restricted ranges. Indeed, based on the College Board norms, the SAT-V and SAT-M scores in the present study ranged from the 2 to 100 percentiles [51]. Moreover, all skew and kurtosis statistics were ≤3.00 and ≥−3.00—a finding that suggests normal univariate distributions for all of the measures.

In addition, the correlations between the predictors and overall SAT scores, which are reported in Table 1, were analogous to those observed in other studies. For instance, the present study’s 0.18 academic self-efficacy-overall SAT correlation was analogous to the 0.15 correlation observed by Robins et al. [24], *z* < 1.00. The present study’s −0.37 performance avoidance-overall SAT scores correlation was analogous to the −0.33 correlation observed by Elliott and McGregor [52], *z* < 1.00. On the other hand, the present study’s −0.40 test anxiety–overall SAT score correlation was not analogous to the −0.31 test anxiety-entrance exam correlation observed by von der Embse et al. [21], *z* = 2.03, *p* < 0.03. However, von der Embse et al.’s −0.31 correlation was based on a variety of entrance exams (e.g., ACT, SAT, state exams) that were administered to students at multiple education levels (primary, middle school, secondary). Thus, it is unclear whether von der Embse et al.’s correlation is lower or higher than one would expect with just the SAT alone. Moreover, although the present study’s test anxiety–overall SAT correlation is significantly different from von der Embse et al.’s −0.31 correlation, the present study’s −0.33 test anxiety–SAT-M and −0.38 test anxiety–SAT-V correlations are not significantly different from von der Embse et al.’s −0.31 correlation, *z* < 1.00 and *z* = 1.50 respectively.

Table 2 reports the means and standard deviations for the measures as a function of ethnicity. As shown in Table 2, for each measure the standard deviations are equivalent for European-American and Hispanic students, which suggests equal variability between the two ethnic groups. Although it is not noted in Table 2, 45.71% of the European-American (i.e., 128/280) and 48% of the Hispanic students (i.e., 84/175) were male.

In addition, the average ages for the two groups of students were 19.29 (*std* = 1.47) and 19.38 (*std* = 1.62) respectively. Two-tailed t-tests, with *p*-values set to 0.005, were computed in order to identify significant differences between the two ethnic groups for each specific measure. This lower significance level was used in order to reduce type 1 errors and was calculated by setting a family-wise alpha, namely dividing alpha by the number of tests (i.e., 0.05/11). Finally, effect sizes (i.e., Cohen’s ds) were calculated for each measure. According to Cohen [53], a *d* = 0.80 is a large effect, a *d* = 0.50 is a moderate effect, and a *d* = 0.20 is small. Asterisks in Table 2 denote measures that have significant ethnic differences.

### 3.3. Comparisons of Means and Correlations between the Ethnic Groups

Consistent with Kobrin et al., [6], the overall SAT, SAT-V, and SAT-M scores of European-American students were all significantly higher than the analogous scores for Hispanic students, 1115.00 (*std* = 134.88), 559.77 (*std* = 81.96), 555.45 (*std* = 74.91) versus 990.11 (*std* = 129.81), 491.26 (*std =* 75.11), and 498.86 (*std* = 71.56) respectively, *min t* (455) = 7.98, *p <* 0.001, *min d =* 0.77. In addition, European-American students’ scores on the measures of knowledge integration, operation span, test anxiety, performance avoidance, highest parent education, family income, and epistemic belief of learning were significantly different from their Hispanic counterparts, *min t* (453) *=* 3.63, *p <* 0.001, *min d =* 0.34. In fact, the only measure without a significant ethnic difference was academic self-efficacy, *t* (453) = 0.45, *p* = 0.66, *d* = 0.04.

Besides examining the means of the predictors for ethnic differences, many of the correlations that included the ethnicity variable were significant. For instance, as Table 1 shows, the three correlations between SAT, SAT-V, and SAT-M scores and ethnicity suggest that Hispanic students have significantly lower overall SAT, SAT-V, and SAT-M scores than do European-American students, *range of r =* −0.35 to −0.42. In addition, with the exception of academic self-efficacy, all the predictors correlated significantly with ethnicity, *range of r* = −0.17 to −0.38, a finding that again suggest that Hispanic students’ performances on all of these predictors differed from their European-American counterparts. Finally, it appears that the *r* = −0.32 highest parent education and *r* = −0.38 family income correlations with ethnicity were significantly stronger than all other predictor-ethnicity correlations, *min t (453)* = 1.91, *p* < 0.05.

### 3.4. Regression Analyses

A regression analysis was completed for each SAT measure. Each regression initially included: (i) the composite measure of cognitive abilities, (ii) ethnicity, (iii) the social/personality predictors: test anxiety, performance avoidance, and academic self-efficacy, (iv) the measure of metacognitive awareness, (v) SES, (vi) the ethnicity X cognitive abilities interaction, (vii) the ethnicity X social/personality predictor interactions: Ethnicity X test anxiety, ethnicity X performance avoidance, ethnicity X academic self-efficacy, (viii) the ethnicity X metacognitive awareness interaction, and (ix) ethnicity X SES interaction. The ethnicity X predictor interactions were included in order to determine whether there were ethnic differences in the contributions of the predictors to overall SAT, SAT-M, and/or SAT-V scores. The ethnicity predictor and the ethnicity X predictor interactions were created by dummy coding European-Americans with an ethnicity of zero (i.e., 0) and Hispanics with an ethnicity of one (i.e., 1). The ethnicity X predictor interactions were not mean centered.

For all three regressions, the composite measure of cognitive abilities was entered into the models first followed by the other main effect predictors (i.e., test anxiety, performance avoidance, academic self-efficacy, metacognitive awareness, and SES) and the ethnicity X predictor interactions. These remaining main effect predictors and ethnicity X predictor interactions were entered into the models in a forward stepwise fashion^5^. This approach allowed the present study to determine the amount of unique variance that non-cognitive predictors (i.e., all predictors except the composite measure of cognitive abilities) contributed to overall SAT, SAT-M, and SAT-V scores after the variance attributed to cognitive abilities was removed. Such information may be important given that researchers have suggested that the SAT is primarily a measure of major cognitive factors such as intelligence [14].

Table 3 reports the significant predictors from these three regressions as well as the main effect predictors for the significant ethnicity X predictor interactions; all other non-significant predictors, including ethnicity X interactions are not reported in Table 3. Column 1 in Table 3 identifies the predictors/ethnicity X predictor interactions and columns 2, 3, and 4 in Table 3 report the estimates, p-values, and unique variance for each predictor/ethnicity X predictor interaction. Because the ethnicity X test anxiety interaction was significant for all three SAT measures, the ethnicity and test anxiety predictors were forced into the regressions even when they were not significant. All other predictors reported in Table 3 were significant, *p* < 0.05. The significant predictors reported in Table 3 were the predictors included in the final models; predictors reported in Table 3 that were not significant and predictors not included in Table 3 were not included in the final models. Finally, the statistics for the predictors reported in Table 3 are the final statistics after non-significant predictors that are not reported in Table 3 were removed.

In general, cognitive abilities, performance avoidance, metacognitive awareness, and SES accounted for significant amounts of unique variance in overall SAT, SAT-M, and SAT-V scores. In addition, the ethnicity X test anxiety interaction accounted for a significant amount of unique variance in all three SAT measures, a finding that indicates the presence of significant ethnic differences. However, none of the other five ethnicity x predictor interaction predictors reached significance. Subsequent power analysis revealed that these non-significant interaction predictors were not a consequence of insufficient N, since the present study exceeds the minimum N of 395, which is necessary to detect a small effect, with an alpha = 0.05 and power set to 0.80 (i.e., the present study included 457 students).

#### 3.4.1. Cognitive, Metacognitive Awareness, Social/Personality, and Socioeconomic Predictors of Overall SAT, SAT-M, and SAT-V

As Table 3, panel (i) shows, the significant predictors accounted for 44.23% of the variance in overall SAT scores: The composite measure of cognitive abilities accounted for 23.89% of this variance and the significant ethnicity X test anxiety, metacognitive awareness, performance avoidance, and SES predictors accounted for the other 19.41% variance. Notably, the ethnicity X test anxiety interaction accounted for more than 60% of the variance that was not cognitive (i.e., 13.23/19.41 = 68.16%).

A similar pattern of results emerged for the SAT-M and SAT-V scores. That is, as Table 3, panel (ii) shows, the significant predictors accounted for 31.03% of the variance in SAT-M scores: The composite measure of cognitive abilities accounted for 16.75% of this variance and the remaining significant predictors of ethnicity X test anxiety, performance avoidance, metacognitive awareness, and SES accounted for the other 14.28% variance. In addition, the ethnicity X test anxiety interaction accounted for more than 60% of the variance that was not cognitive (i.e., 9.77/14.28 = 68.42%).

Similarly, as Table 3 panel (iii) shows, the significant predictors accounted for 37.96% of the variance in SAT-V scores: The composite measure of cognitive abilities accounted for 20.65% of this variance and the remaining significant predictors of ethnicity X test anxiety, metacognitive awareness, performance avoidance, SES, and test anxiety accounted for the other 17.31% variance. However, the significant main effect for test anxiety should not be interpreted in isolation because test anxiety makes an additional contribution to SAT-V scores via the ethnicity X test anxiety interaction. Finally, the ethnicity X test anxiety interaction accounted for more than 60% of the variance that was not cognitive (i.e., 11.14/17.31 = 64.36%).

#### 3.4.2. Ethnic Differences in the Predictors of Overall SAT, SAT-M, and SAT-V

With respect to ethnic differences in overall SAT, SAT-M, and SAT-V scores, the most notable predictor was the ethnicity X test anxiety interaction because it suggests that test anxiety’s contributions to the three SAT measures vary as a function of ethnicity. Figure 1 depicts the ethnicity X test anxiety interaction for overall SAT scores and Figure 2 depicts the ethnicity X test anxiety interaction and test anxiety main effect for SAT-V scores. A figure is not provided for the SAT-M scores because the pattern of results is identical to the pattern of results for the overall SAT scores. In both figures, the solid line represents the European-American students’ SAT scores as a function of low and high test anxiety and the dashed line represents the Hispanic students’ SAT scores as a function of low and high test anxiety. The low and high anxiety scores that are used in the figures are the low and high test anxiety scores reported in this study, 2 and 33 respectively.

As Figure 1 shows, whereas test anxiety did not contribute to European-American students’ overall SAT scores, test anxiety contributed negatively to Hispanic students’ overall SAT scores. When Hispanic students’ test anxiety scores were low (e.g., a score of 2), test anxiety had a minor negative influence on Hispanic students’ overall SAT scores. On the other hand, when Hispanic students’ test anxiety scores were high (e.g., a score of 33), test anxiety had a much larger negative influence on Hispanic students’ overall SAT scores.

The pattern of results was similar for SAT-M scores. Whereas test anxiety did not contribute to European-American students’ SAT-M scores, test anxiety contributed negatively to Hispanic students’ scores. When test anxiety scores were low (e.g., 2), test anxiety had a minor negative influence on Hispanic students’ SAT-M scores but when test anxiety scores were high (e.g., 33), test anxiety had a much larger negative influence on Hispanic students’ SAT-M scores.

For SAT-V scores, interpreting the contributions of test anxiety is more complicated because both the significant main effect of test anxiety and the ethnicity X test anxiety interaction must be interpreted together. Whereas the main effect of test anxiety indicates that test anxiety contributed negatively to both European-American and Hispanic students’ SAT-V scores, the ethnicity X test anxiety interaction indicates that test anxiety makes an additional negative contribution to Hispanic students’ SAT-V scores but not to European-American students’ SAT-V scores. In other words, although test anxiety makes a deleterious contribution to both European-American and Hispanic students’ SAT-V scores, its deleterious contribution to Hispanic students’ SAT-V scores is much more profound.

As Figure 2 shows, although European-American students’ SAT-V scores decreased slightly when their test anxiety scores were low, their SAT-V scores decreased more when their test anxiety scores were high^6^. In contrast, when Hispanic students’ test anxiety scores were low, their SAT-V scores decreased slightly more than they did for European-American students with low test anxiety scores. More importantly though, when Hispanic students’ test anxiety scores were high, their SAT-V scores decreased much more; indeed, substantially more than they did for European-American students with high test anxiety.

## 4. Discussion

The present study used regression analyses in order to determine the contributions of cognitive (i.e., working memory, knowledge integration), metacognitive awareness, social/ personality (i.e., test anxiety, performance-avoidance goals, academic self-efficacy) and SES factors to European-American and Hispanic students’ overall SAT, SAT-M, and SAT-V scores. That is, do all of these factors contribute to the SAT scores of European-American versus Hispanic students to the same extent? The present study showed that cognitive and non-cognitive factors contribute to overall SAT, SAT-M, and SAT-V scores. More interestingly though, the ethnicity X test anxiety interaction was also significant, accounting for more unique variance in the three SAT measures than any other significant predictor that was not cognitive.

The finding that the ethnicity X test anxiety interaction was significant is an important one because it suggests ethnic differences in the contributions of test anxiety to overall SAT, SAT-M, and SAT-V scores. Indeed, the present study observed that whereas test anxiety made no significant contribution to European-American students’ overall SAT scores, test anxiety made a negative contribution to Hispanic students’ overall SAT scores, especially when their test anxiety scores were high. A similar pattern of results emerged for SAT-M scores. Whereas test anxiety made no contribution to European-American students’ SAT-M scores, it negatively contributed to Hispanic students’ scores. Again, this negative contribution was particularly profound when Hispanic students’ test anxiety scores were high.

For SAT-V scores, interpreting the negative contribution of test anxiety was more complicated because besides the significant ethnicity X test anxiety interaction, the main effect of test anxiety was also significant. Unlike overall SAT and SAT-M scores, test anxiety made a negative contribution to European-American SAT-V scores; although this contribution was a minor-to-modest one (i.e., modest/small decreases in SAT-V scores with higher test anxiety scores resulting in greater decreases). In contrast, for Hispanic students when test anxiety scores were low their SAT-V scores decreased slightly more than they did for European-American students. Moreover, when their test anxiety scores were high, Hispanic students’ SAT-V scores decreased much more; indeed, substantially more than the SAT-V scores for European-American students with high test anxiety.

The present study’s finding that cognitive abilities account for variance in overall SAT scores supports the findings of other researchers, who also showed that cognitive abilities are predictors of overall SAT scores. Indeed, consistent with Engle and colleagues [18] the present results showed that cognitive abilities account for a significant amount of variance in SAT scores (i.e., *r* = 0.49). However, the present results also extend the findings of Engle and colleagues [18] in two important ways. First, the present study shows that other non-cognitive predictors, such as performance-avoidance, metacognitive awareness, and SES, account for unique variance in overall SAT, SAT-M, and SAT-V scores, even after the variance attributed to cognitive abilities has been removed. Second, the present study identifies at least one source of ethnic differences in the three SAT measures, namely test anxiety.

Besides the ethnicity X test anxiety interaction, the negative contributions of the non-cognitive predictors (i.e., significant main effects) to overall SAT, SAT-M, and SAT-V scores are also noteworthy, especially for Hispanic students. As noted in Table 2, with the exception of academic self-efficacy, all of the non-cognitive predictors had significant ethnic differences, range of *d* = 0.34 to 0.82, with Hispanic students having the poorer performance in all instances. What this means is that, Hispanic students, in general, will have lower overall SAT, SAT-M, and SAT-V scores than their European-American counterparts—not just because of ethnic differences (i.e., ethnicity X test anxiety)—but because of poorer performances on the significant non-cognitive predictors of overall SAT, SAT-M, and SAT-V scores (i.e., metacognitive awareness, performance avoidance, and SES).

In addition, the finding that academic self-efficacy had significant correlations with overall SAT, SAT-M, and SAT-V scores but failed to be a significant predictor of any of these SAT measures, partially corroborates the research of Judge, Erez, Bono, and Thoresen [54]. Across four studies, these authors [54] observed that measures of self-esteem, neuroticism, locus of control, and generalized self-efficacy were not only strongly related to each other but that the relationships among these measures could also be explained by a single latent factor. Moreover, these authors [54] reported poor discriminant validity and little incremental validity for the measures of these four constructs. Consistent with Judge et al. [54], the present study also observed that academic self-efficacy accounted for no unique variance in overall SAT, SAT-M, and SAT-V scores, once the variance attributed to cognitive and non-cognitive factors had been removed—a finding that also provides little evidence of discriminant and incremental validity for the present study’s measure of academic self-efficacy.

From a theoretical perspective, the present results are informative. For instance, the present results suggest that it is plausible that no single factor/construct predicts the SAT. Rather, it appears that the SAT is a complex construct, predicted by many factors, including cognitive, metacognitive awareness, social, personality, and socioeconomic family background factors [14]. Indeed, the present study adds to the complexity of the overall SAT, SAT-M, and SAT-V by showing that the significance and/or relative contribution of test anxiety to SAT, SAT-V, and SAT-M varies as a function of the ethnic background of a student.

Of course, there is always the possibility that it is not test anxiety that is causing low SAT scores but rather low SAT scores that are causing a high anxiety score. In other words, poor performance on the SAT permanently changes a student’s anxiety score. Such a possibility is consistent with the *deficit model,* which states that inadequate or deficit learning/test-taking skills negatively affects measures of academic achievement (i.e., learning/test-taking skills → academic achievement → test anxiety) [55]. In order to test for this possibility, future research might wish to follow, longitudinally, students who have good and poor learning/test-taking skills and monitor how these skills influence academic performance and test anxiety.

The present results also serve as a foundation for future research. For instance, it is possible that other factors, such as self-handicapping, account for unique differences in SAT performances. In addition, although Koenig, Frey, and Detterman [56] observed that general intelligence was a strong predictor of scores on the Academic College Test (i.e., ACT), another academic admissions test that is used as an alternative to the SAT, these researchers did not include measures of social/personality factors, such as test anxiety, performance avoidance, academic self-efficacy, and/or socioeconomic family background. Nor did Frey and colleagues [56] determine whether the contributions of general intelligence varied as a function of the ethnicity of their participants. By considering these other factors, future research may very well observe that the prediction of ACT scores is analogous to that of SAT scores. Another possible avenue for future research is to determine whether the contributions of the predictors to SAT/ACT vary as a function of gender. Hannon [13], for instance, showed that social/personality factors (i.e., performance avoidance, test anxiety) but not cognitive factors (i.e., working memory, knowledge integration) accounted for 100% of the gender differences that are routinely observed with the SAT; see [5] for a discussion of gender differences in the SAT. However, Hannon [13] did not examine the relative contributions of social/personality factors to females versus males’ SAT scores. Consequently, it is unclear whether social/personality factors predict females versus males’ SAT scores to the same extent. Finally, future research might consider a propensity score matching approach when considering ethnic and/or gender differences in SAT scores. For example, ethnic groups might be matched on SAT scores, test anxiety scores, or perhaps even general intelligence scores.

The present study also has a number of limitations. For instance, the present study is based on two sets of data following selection at one university rather than multiple universities; consequently, it is unclear whether the present results generalize to students located in other parts of the U.S. The present study also did not include other minority groups, such as Asian and African-American students and so it is unknown whether these two ethnic groups have SAT predictor profiles that are similar or different from European-American and Hispanic students. The results of the present study are also based on specific measures of cognitive abilities, test anxiety, performance avoidance, and metacognitive awareness. Thus, it is possible that the present results over- or under-estimate the influences of these predictors. The present study did not include measures of fluid intelligence, such as Raven’s Advanced Progressive Matrices or Cattell’s Culture Fair Test. Thus, it is possible that fluid intelligence might account for additional variance in the three SAT measures. In addition, the present study does not consider the relationships among the predictors. Such an approach is necessary in order to develop a theory as to what the SAT is measuring. In addition, because no pre-existing theory explains the SAT using cognitive, metacognitive, social/personality, and socio-economic factors, the present study chose to use forward stepwise regression analysis after the cognitive factor was entered into the SAT models. Although stepwise regression analysis is suitable for exploratory purposes, like the present study, stepwise regression analysis not only limits the many possible models that can be tested but it also potentially underestimates certain combinations of variables. Given the strong ethnicity X test anxiety interactions for all three SAT measures, it is also possible that measurement invariance was violated for the test anxiety measure and/or the three SAT measures. Finally, because assessment of the predictors took place months after students completed the SAT, the present study cannot, unfortunately, establish causality.

In summary, the present study showed that cognitive and non-cognitive factors contribute to overall SAT, SAT-M, and SAT-V scores. In addition, the ethnicity X test anxiety interaction was a significant predictor for all three SAT measures, a finding that suggests ethnic differences in the contributions of test anxiety to overall SAT, SAT-M, and SAT-V scores. For European-American students, test anxiety had no influence on overall SAT and SAT-M scores, whereas for Hispanic students test anxiety had a negative influence on overall SAT and SAT-M scores. For SAT-V scores, interpreting the ethnicity X test anxiety interaction was more complicated because both the significant main effect of test anxiety and the ethnicity X test anxiety interaction must be interpreted together. Whereas test anxiety negatively influenced European-Americans’ SAT-V scores, this negative influence was less than the influence it had on Hispanic students’ SAT-V scores. Indeed, for Hispanic students with high test anxiety, this negative influence was profound.

## Figures and Tables

**Table 1:** Correlations and Descriptive Statistics among Ethnicity, and the Measures of Academic Achievement (i.e., SAT-V, SAT-M, and SAT), Test Anxiety, Performance Avoidance, Academic Self-efficacy, Highest Parent Education, Family Income, and Metacognitive Awareness (i.e., Epistemic Belief of Learning) (*n =* 455).

	1	2	3	4	5	6	7	8	9	10	11	12
1. Ethnicity ^a^	--	−0.39 *	−0.35 *	−0.42 *	0.20 *	0.18 *	−0.02	−0.17 *	−0.18 *	0.21 *	−0.32 *	−0.38 *
2. SAT-V		--	0.58 *	0.90 *	−0.38 *	−0.32 *	0.18 *	0.36 *	0.36 *	−0.35 *	0.29 *	0.17 *
3. SAT-M			--	0.88 *	−0.33 *	−0.34 *	0.14 *	0.32 *	0.33 *	−0.27 *	0.20 *	0.20 *
4. Overall SAT				--	−0.40 *	−0.37 *	0.18 *	0.38 *	0.38 *	−0.35 *	0.28 *	0.21 *
5. Test anxiety					--	0.55 *	−0.53 *	−0.22 *	−0.20 *	0.22 *	−0.15 *	−0.05
6. Performance avoidance						--	−0.33 *	−0.22 *	−0.12 *	0.23 *	−0.14 *	−0.03
7. Academic self-efficacy							--	0.17 *	0.07	−0.15 *	0.6	0.01
8. Knowledge integration								--	0.24 *	−0.16 *	0.16 *	0.00
9. Operation span									--	−0.22 *	0.16 *	0.08
10. Epistemic belief of learning										--	−0.13 *	−0.13 *
11. Highest parent education											--	0.42 *
12. Family income												--
Mean	0.39	533.42	533.68	1067.00	15.23	4.56	51.63	26.62	73.43	34.32	6.80	4.23
Standard deviation	0.49	86.05	78.55	146.07	7.57	1.12	8.14	5.25	12.04	5.10	2.42	1.71
Skewness	0.48	0.31	−0.02	0.56	0.35	−0.45	−0.29	−0.33	−0.15	−0.15	−1.16	−0.36
Kurtosis	−1.78	−0.18	−0.24	−0.33	−0.81	−0.07	−0.08	−0.72	−0.57	−0.34	0.08	−0.76
Lowest score	0.00	330.00	320.00	690.00	2.00	1.00	29.00	13.00	37.00	20.00	1.00	0.00
Highest score	1.00	800.00	740.00	1450.00	33.00	7.00	70.00	36.00	98.00	47.00	9.00	7.00
Maximum score	1.00	800.00	800.00	1600.00	37.00	7.00	70.00	36.00	100.00	60.00	9.00	7.00

*Note.* * *p* < 0.05. ^a^ For ethnicity, European-Americans = 0 and Hispanics = 1; for example, the −0.35 correlation between ethnicity and SAT-M means that European-American students have higher SAT-M scores than Hispanic students.

**Table 2:** Descriptive Statistics for SAT, Cognitive, Social/Personality, Socioeconomic Family Background, and Metacognitive Awareness Measures as a Function of Ethnicity.

	European-Americans (*n* = 280)	Hispanics (*n* = 175)	Effect Size
***SAT***			
Overall SAT *	1115.00 (134.88)	990.11 (129.81)	*d* = 0.94
SAT-V *	559.77 (81.96)	491.26 (75.11)	*d* = 0.87
SAT-M *	555.45 (74.91)	498.86 (71.56)	*d* = 0.77
***Cognitive Measures***			
Knowledge integration *	27.31 (5.13)	25.50 (5.25)	*d* = 0.34
Operation span *	75.11 (11.04)	70.75 (13.07)	*d* = 0.36
***Social/Personality Measures***			
Test anxiety *	14.01 (7.41)	17.18 (7.42)	*d* = 0.42
Performance-avoidance goals *	4.41 (1.14)	4.82 (1.05)	*d* = 0.37
Academic self-efficacy	51.76 (8.13)	51.41 (8.16)	*d* = 0.04
***Socioeconomic Family Background Measures***			
Highest parent education *	7.41 (1.93)	5.82 (2.78)	*d* = 0.66
Family income *	4.74 (1.48)	3.42 (1.74)	*d* = 0.82
***Metacognitive Awareness Measure***			
Epistemic belief of learning *	33.47 (5.31)	35.67 (4.43)	*d* = 0.45

*Note.* Standard deviations are in brackets. * indicates a significant difference between the groups, all significant *p’*s < 0.001.

**Table 3:** Regression Models Predicting Overall SAT, SAT-M, and SAT-V Scores. The Composite Measure of Cognitive Abilities entered into the Models First and the other Predictors entered in a Forward Stepwise Fashion.

			Relative Importance	Beta
Predictor	Estimate	*p*	(Variance Decomposition)	Coefficient

**(i) Overall SAT**				
Intercept	1342.62	0.0001		
Cognitive abilities	30.84	0.0001	0.2389	0.32
Ethnicity X test anxiety	−4.04	0.0001	0.1323	−0.20
Performance avoidance	−22.41	0.0001	0.0321	−0.14
Metacognitive awareness	−4.27	0.0001	0.0213	−0.15
SES	8.61	0.0101	0.0084	0.15
Test anxiety	−2.30	0.0742	*0*.0040 *(not significant)*	−0.07
Ethnicity	−39.17	0.1307	0.0029 *(not significant)*	−0.05
*Total variance from significant predictors:*			0.4330	

**(ii) SAT-M**				
Intercept	651.67	0.0001		
Cognitive abilities	13.85	0.0001	0.1675	0.28
Ethnicity X test anxiety	−1.87	0.0001	0.0977	−0.22
Performance avoidance	−12.32	0.0001	0.0310	−0.18
Metacognitive awareness	−1.44	0.0258	0.0085	−0.09
SES	3.78	0.0465	0.0056	0.09
Test anxiety	−0.85	0.1870	0.0020 *(not significant)*	−0.06
Ethnicity	−9.72	0.5308	0.0014 *(not significant)*	−0.04
*Total variance from significant predictors:*			0.3103	

**(iii) SAT-V**				
Intercept	689.97	0.0001		
Cognitive abilities	16.37	0.0001	0.2065	0.30
Ethnicity X test anxiety ^a^	−1.87	0.0001	0.1114	−0.20
Metacognitive awareness	−2.78	0.0001	0.0331	−0.16
Performance avoidance	−6.75	0.0500	0.0144	0.12
SES	5.26	0.0110	0.0079	0.10
Test anxiety ^a^	−1.18	0.0339	0.0063	0.10
Ethnicity	−27.67	0.0841	0.0041 *(not significant)*	−0.07
*Total variance from significant predictors:*			0.3796	

*Note*. ^a^ The test anxiety main effect and ethnicity X test anxiety interaction should be interpreted together. The test anxiety and ethnicity main effects are reported above because the ethnicity X test anxiety interactions were significant. However, the final models did not include these main effects and interactions if they were not significant.

## Footnotes

1 Metacognitive awareness was classified as non-cognitive because it is a measure that assesses belief about learning rather than a measure of a specific cognitive process/skill.

2 The terms Hispanic, African-American, and European-American are used in the context of US culture. It is acknowledged that these terms are interpreted differently in other cultures.

3 The standard deviations ranged from 99 to 106 for the 20-year period reported above.

4 In their first study, Frey and Detterman [12] noted a non-linear relationship between SAT scores and intelligence.

5 The default *p*-value that the stepwise regression procedure in SAS uses to add a predictor into a model is *p* < 0.15. The default *p*-value for removing a predictor is *p* > 0.15. Because predictors were either significant with a *p* < 0.05 or non-significant with a *p* > 0.15, there was no need to alter these default values.

6 The ethnicity X test anxiety interaction makes no contribution to European-American SAT scores because ethnicity is coded as a zero for European-American students. Therefore, −1.82 * 0 * 2 = 0 and −1.82 * 0 * 33 = 0.
